# Improved On-Site Characterization of Arsenic in Gypsum from Waste Plasterboards Using Smart Devices

**DOI:** 10.3390/ma15072446

**Published:** 2022-03-26

**Authors:** Masamoto Tafu, Juna Nakamura, Momoka Tanii, Saori Takamatsu, Atsushi Manaka

**Affiliations:** 1 Department of Applied Chemistry and Chemical Engineering, National Institute of Technology, Toyama College, Toyama 939-8630, Japan; stakamatsu@nc-toyama.ac.jp (S.T.); manaka@nc-toyama.ac.jp (A.M.); 2 ECOdesign Engineering Program, Advanced Course, National Institute of Technology, Toyama College, Toyama 939-8630, Japan; h1611334@mailg.nc-toyama.ac.jp (J.N.); momoka19990828@icloud.com (M.T.)

**Keywords:** plasterboard, arsenic, recycling, on-site determination

## Abstract

The impurities in waste plasterboards, a product of ethical demolition, are a serious problem for their recycling. Plasterboards, the wall materials used in old buildings, are often recycled into gypsum powder for various applications, including ground stabilization. However, this powder contains various chemical impurities from the original production process of the gypsum itself, and such impurities pose a risk of polluting the surrounding soil. Here, we present a simple method for verifying the presence of arsenic, a harmful element in recycled gypsum that is suitable for use at demolition sites. First, we developed a simple pretreatment method using a cation-exchange resin to dissolve insoluble gypsum suspended in water by exploiting a chemical equilibrium shift, and we estimated the quantity suitable for releasing the arsenic from arsenic-containing gypsum. This pretreated solution could then be tested with a conventional arsenic test kit by observing the color changes in the test paper using the image sensor of a smart device. This simple method could determine a wide range of arsenic quantities in the gypsum, which would be helpful for monitoring arsenic in recycled gypsum powder, thereby supporting the development of a safe circular economy for waste plasterboards.

## 1. Introduction

**Figure 1:**
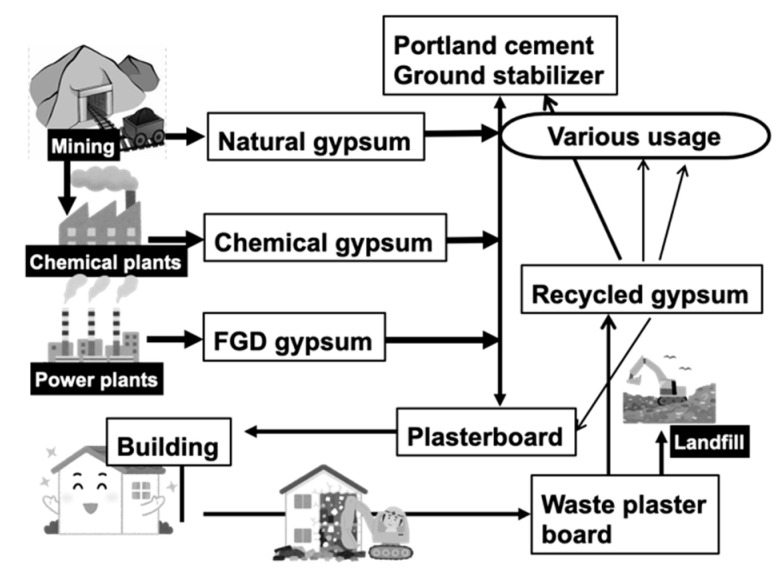
Material flow of gypsum in Japan. FGD: flue gas desulfurization.

Plasterboards consisting of solidified gypsum (calcium sulfate dihydrate, CaSO_4_·2H_2_O) between paper sheets are widely used as wall materials in houses constructed using the 2 × 4 (two-by-four) method. In Japan, the lifetime of houses is approximately 40 years, and when houses are demolished, the plasterboards are collected for recycling. Specifically, the reclaimed gypsum is pulverized, treated, and used in new plasterboards. However, this recycling process is limited, and most gypsum in plasterboards is derived from mining (natural gypsum) as well as the byproducts of various chemical plant processes (chemical gypsum) and flue gas desulfurization (FGD), as shown in Figure 1. Specifically, chemical gypsum originates from phosphate and fluoride production and smelting, whereas FGD gypsum is a byproduct of thermal power plants using coal and heavy oil. Moreover, it potentially contains chemical impurities, including fluoride, arsenic, and cadmium, derived from the raw materials used in these chemical processes. As shown in this figure, gypsum is also is widely used as a component of Portland cement, as well as a ground stabilizer to improve ground hardness, which has been exhaustively studied. However, because gypsum in plasterboards is supplied from various sources, the recycled gypsum from waste plasterboards poses a risk of soil pollution by potentially releasing fluoride, arsenic, and cadmium into the surrounding soil.

In particular, the arsenic in waste gypsum has severe environmental effects. As a hazardous waste product of the metallurgical industry, arsenic-bearing gypsum (ABG) is derived from the lime neutralization of waste acid liquor [1,2]. Some amount of ABG was used in plasterboards in Japan from 1973 to 1997, and in 2017, approximately 1.5 × 10^4^ tons of ABG were separated from abandoned buildings in Japan [3]. Waste plasterboards containing ABG must be carefully collected during demolition, and ABG-containing waste plasterboards must be identified to safely recycle gypsum for ground stabilization to avoid polluting the soil. Ideally, this identification is carried out directly at construction sites, which could lead to the development of a safer circular economy for recycled gypsum from waste plasterboards.

One on-site determination method for analyzing the arsenic in gypsum is X-ray fluorescence (XRF) [4,5], but this method requires skilled handling and/or a license to operate the radiation apparatus. To overcome this problem, we focused on adapting facile commercial test kits for determining the arsenic contents in an aqueous solution, a method that can be employed in the field. Because of the low solubility of gypsum, determining its arsenic levels requires a pretreatment to dissolve the gypsum into a homogeneous solution through a pyrolysis process involving harmful chemicals (such as hydrochloric acid [6] or perchloric acid [4]). After pretreatment, arsenic is released into the solution in a form suitable for detection, and conventional analytical methods can then be applied. We previously demonstrated that gypsum was easily dissolved in water containing cation- and anion-exchange resins and that the fluoride content in gypsum could be successfully determined in the resulting solution using a simple colorimetric method [7]. Based on these earlier findings, we hypothesized that the arsenic in waste gypsum could also be released by a pretreatment technique using only a cation-exchange resin because arsenic forms arsenate anions.

Therefore, in this study, we aimed to develop a simple pretreatment method for determining the arsenic levels in the gypsum from waste plasterboards to facilitate its use as a ground stabilizer. We adopted the following approach. First, we determined the suitable quantity of cation-exchange resin required to dissolve gypsum and release the arsenic it contains into water. The volume of arsenic in the resultant solution could then be determined using a conventional arsenic determination test kit based on Gutzeit′s method. We also endeavored to interpret the color change in the test paper from the arsenic test kit based on the concentration of arsenic using data from the image sensors in smart devices, such as smartphones and/or tablets. The results suggest that the proposed method can rapidly determine the amount of arsenic in gypsum. We expect this innovative technique to facilitate the monitoring of harmful pollutants in recycled gypsum powder obtained from waste plasterboards for environmental safety.

## 2. Materials and Methods

### 2.1. Materials and Samples

First, we prepared arsenic-containing gypsum samples for characterization instead of using existing ABG from waste plasterboards. Calcium sulfate dihydrate (FUJI FILM Wako Pure Chemical, Bellwood Rd, VA, USA) was used to prepare this arsenic-containing gypsum by mixing 0.3 g of reagent gypsum and 10 cm^3^ of aqueous solutions containing various amounts of sodium arsenite. Each mixture was then ultrasonicated for 5 min and dried in a convection oven at 80 °C for 24 h. The water used in all experiments was prepared via ion exchange and ultrapurification using a Milli-Q water purification system (Milli-Q A10, Merck-Millipore, Burlington, MA, USA).

### 2.2. Dissolution of Gypsum by Cation-Exchange Resin

A total of 300 mg of the arsenic-containing gypsum samples was mixed with 20 cm^3^ of water, and various amounts of a cation-exchange resin were added (Amberlite IR120 H, DuPont Water Solutions, Wilmington, DE, USA). The mixture was shaken at 200 strokes per minute for 5 min using a reciprocal shaker. The temperature was adjusted to 298 K. The liquid phase was separated via pressure filtration through a cartridge membrane filter (pore size: 0.45 μm). The amount of gypsum dissolved in the water was analyzed by determining the calcium and sulfur content using inductively coupled plasma atomic emission spectrometry (ICP-AES, 720ES, Agilent Technologies, Inc., Santa Clara, CA, USA) with argon plasma.

### 2.3. Determination of Arsenic Content in the Gypsum

In order to measure the amount of arsenic present in an aqueous solution, two types of arsenic test kits based on Gutzeit’s colorimetric method for lower and higher contents (MQuant Arsenic tests, model 1.01747 and 1.17927, respectively, Merck KgaA, Darmstadt, Germany) were selected. The arsenic-containing gypsum samples were dissolved by using the method described above. After the pretreatment, the obtained water samples were tested using the arsenic test kits, which indicate the arsenic contents through changes in the color of the test paper. This color change was determined using the image sensor of a tablet device (ZenPad 8.0, ASUSTeK Computer, Taipei, Taiwan).

In order to confirm these results, the volume of arsenic released from the arsenic-containing gypsum samples was also characterized by ICP-AES, as follows: each sample was mixed with water, and the cation-exchange resin using the method above, and the arsenic content in the obtained solution was analyzed. The determination limit of arsenic by ICP-AES was approximately 0.05 mg/L.

## 3. Results and Discussion

### 3.1. Suitable Volume of Cation-Exchange Resin for Gypsum Dissolution

**Figure 2:**
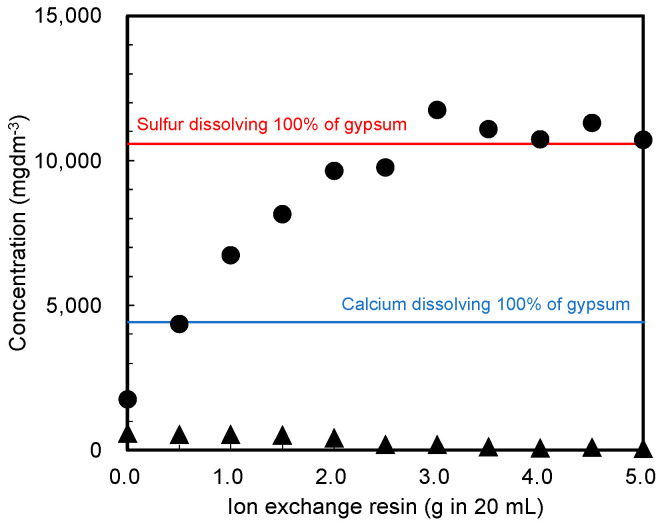
Sulfur and calcium concentrations in the water sample, as measured by ICP-AES after treating the gypsum reagent with a cation-exchange resin. Closed circles (●): sulfur concentration, triangles (▲): calcium concentration. Colored lines: sulfur (red) or calcium (blue) after dissolving 100% of gypsum in water using the resin.

First, the required amount of cation-exchange resin that adequately dissolves gypsum was determined. Figure 2 shows the sulfur and calcium concentrations in water, as measured by ICP-AES after treating the pristine gypsum reagent with the cation-exchange resin. Because of the low solubility of gypsum in water, the calcium and sulfur concentrations after mixing them in water without the cation-exchange resin differed from the values obtained by dissolving all the gypsum in water using the resin (blue and red lines in the figure, respectively). Specifically, adding the cation-exchange resin increased the sulfur concentration and decreased the calcium concentration. This phenomenon indicates that shifting the chemical equilibrium (Equation (1)) to the right successfully dissolved the gypsum in water, which was attributed to a decrease in the calcium concentration as a result of using the ion-exchange resin (Equation (2)).

CaSO_4_ · 2H_2_O ⇌ Ca^2+^ + SO_4_^2−^ + 2H_2_O (1)

R-H^+^ + Ca^2+^ → [R-Ca^2+^]^+^ (2)

In Equation (2), R represents the cation-exchange resin. The experimental results demonstrated that 3.0 g or more of the cation-exchange resin in 20 cm^3^ of water was required to dissolve 0.3 g of gypsum reagent.

**Figure 3:**
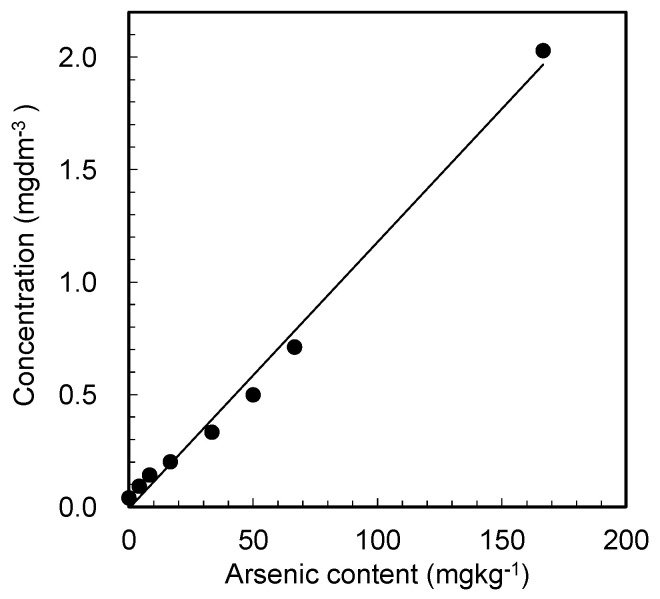
Arsenic concentrations in the water sample, as measured by ICP-AES, after treating the suspensions of the arsenic-containing gypsum samples with a cation-exchange resin as a function of the known arsenic content in the prepared samples. Experimental condition: gypsum: 0.3 g, cation-exchange resin: 4 g in 20 mL of water.

The release of arsenic from the gypsum sample was then examined under the pretreatment conditions. In this study, we prepared arsenic-containing gypsum samples with predetermined amounts of arsenic instead of using existing ABG; thus, the arsenic contents were known and did not require further determination. Figure 3 shows the change in the arsenic concentration in water, as determined by ICP-AES after dissolving the gypsum samples containing various amounts of arsenic.

Figure 3 shows a strong linear relationship between the arsenic contents in the gypsum sample and the arsenic concentration in the treated water. Evidently, the arsenic in the gypsum is successfully released into the water after the cation-exchange resin pretreatment, thus eliminating the need for conventional treatment techniques that employ harmful chemicals. However, the amounts of arsenic released into the treated water were slightly lower than the values estimated from the arsenic used to prepare the gypsum samples. This result suggests that some arsenic ions were adsorbed on the ion-exchange resin. The pretreatment conditions, including the selection of ion-exchange resin, must therefore be optimized in future research.

### 3.2. Improved Determination of Arsenic Concentration Using Conventional Tests and Image Processing

**Figure 4:**
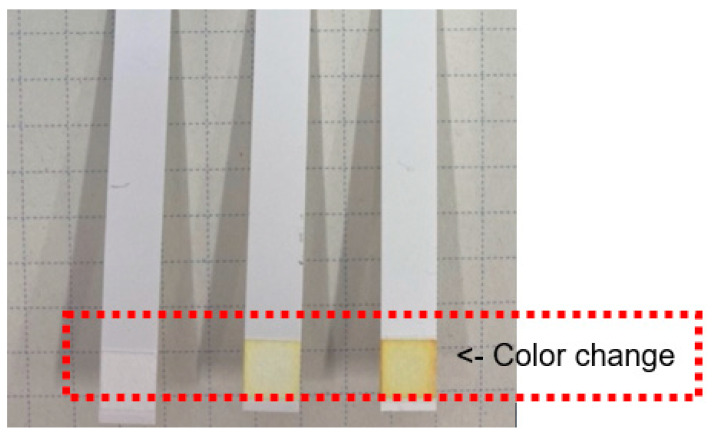
Photograph of the test paper for higher arsenic contents in the presence of different levels of arsenic in the water samples.

**Figure 5:**
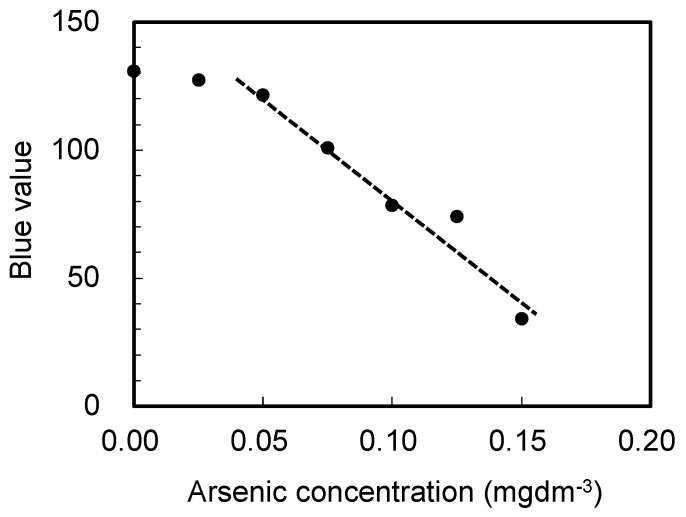
Change in the blue value of the image sensor as a function of the arsenic concentration in the solution.

Next, the amount of arsenic was determined using the arsenic test kit for higher arsenic content by analyzing the arsenic content in the solution obtained after the resin pretreatment, as described in the previous section. Figure 4 shows a photograph of the test paper from the kit after detecting arsenic in different water samples. As shown in the figure, the color change is not easily recognizable without skilled observation. In order to better quantify these results, changes in the output signal from a tablet image sensor after capturing images of the test paper color were plotted as a function of the arsenic concentration, as shown in Figure 5. Evidently, the blue output value from the image sensor strongly correlates with the arsenic content in the solution over a concentration range from 0.05 to 0.15 mgdm^−3^. A similar relationship was obtained using the test kit for lower arsenic contents (data not shown).

**Figure 6:**
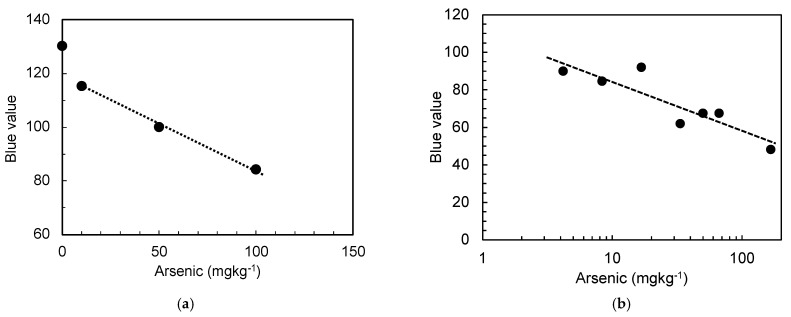
Arsenic levels in arsenic-containing gypsum, as determined using the proposed method, using the test kits for (**a**) high; (**b**) low arsenic contents.

Based on these findings, we further investigated the arsenic levels in the simulated arsenic-containing gypsum. First, each gypsum sample was dissolved in water containing the cation-exchange resin, and the obtained solutions were tested using the arsenic test kit and the tablet for imaging, as described above. The results shown in Figure 6 indicate that the imaging with the tablet enables accurate determination of the arsenic concentration in the solution over the range of 10–100 mg kg^−1^ (Figure 6a) and 4–80 mg kg^−1^ (Figure 6b) using the arsenic test kits for higher and lower arsenic contents, respectively. Based on the results of the kit for lower contents, the arsenic content was thereafter determined using the arsenic test kit for higher arsenic contents, as the results obtained from the arsenic test kit for lower arsenic contents were quantitatively limited. However, these results indicated a higher sensitivity for detecting low arsenic contents in the gypsum.

### 3.3. Benefits of the Study Results in Gypsum Recycling

The results of this study were used to evaluate the benefits of recycling gypsum. Certain properties of gypsum make it suitable for use as a fertilizer [8]; therefore, recycled gypsum from waste plasterboards could be used in agricultural applications if the impurity concentrations are controlled. Because arsenic is harmful to agricultural activities [9], arsenic-containing gypsum should not be recycled for this purpose to avoid soil pollution. However, conventional methods for monitoring arsenic require specific analytical methods, skills, and equipment and involve lengthy processes. Consequently, it can be difficult to determine the volume of arsenic-containing gypsum in waste plasterboards at intermediate waste treatment facilities. Indeed, for the safe recycling of gypsum, determining the presence or absence of arsenic is more significant than quantifying the exact amount of arsenic present. The results described in the previous section suggest that arsenic-bearing gypsum can be easily identified using simplified pretreatment and conventional arsenic test kits. Further, this novel, simple method can be used for the on-site determination of the arsenic content in waste gypsum. Our results could, therefore, be useful in identifying ABG before accepting it for further reprocessing based on its arsenic content.

Fluoride is also an important impurity in waste plasterboards. We previously reported a method for the on-site determination of the fluoride content in waste gypsum by pretreatment with cation- and anion-exchange resins [7]. We also developed a simplified stabilization method for fluoride in gypsum by adding dicalcium phosphate dihydrate (DCPD, CaHPO_4_·2H_2_O) [10,11] using the transformation reaction of DCPD to stable fluorapatite (FAp, Ca_10_(PO_4_)_6_F_2_) [12].

**Figure 7:**
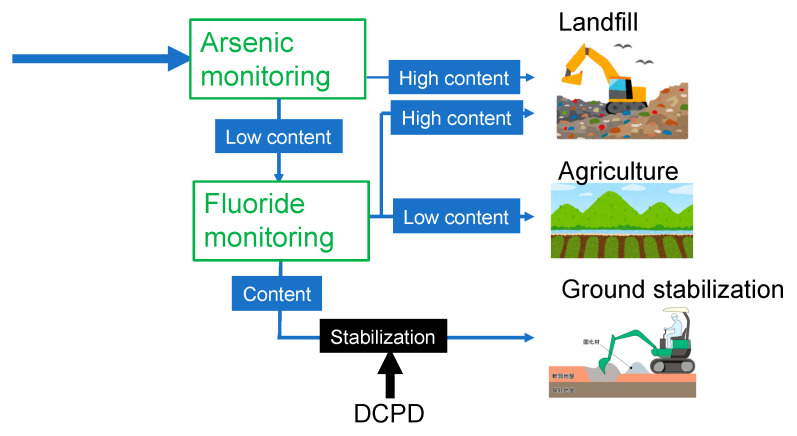
Potential advantages of our findings for the safe reprocessing of recycled gypsum obtained from waste plasterboards. DCPD: dicalcium phosphate dihydrate.

The flowchart in Figure 7 shows the benefits of our combined findings in making gypsum safe for various recycling applications. If the arsenic content is sufficiently low, the gypsum can be saved from being landfilled, but the fluoride content must be checked. If the fluoride content is also sufficiently low, the gypsum can be used in agricultural applications. Alternatively, the fluoride can be stabilized using DCPD, making the gypsum suitable for ground stabilization. Thus, our approach leads to a reduction in the amount of waste gypsum disposed of in landfills, safer use of recycled gypsum in agricultural applications, and the efficient use of DCPD to stabilize fluoride in the recycled gypsum used for ground stabilization, which could prevent the release of fluoride into the surrounding soil.

## 4. Conclusions

The results of this study suggest that the arsenic content in gypsum recycled from waste plasterboards could be determined via a pretreatment method employing an ion-exchange resin, which facilitates the release of arsenic into the solution. Then, the results of conventional colorimetric arsenic test kits can be monitored using a tablet to better quantify the arsenic concentration.

The key points can be summarized as follows:

(1) Although gypsum is a stable compound in water, its solubility was sufficiently enhanced by adding a cation-exchange resin.
(2) Using our proposed method, we accurately determined the arsenic concentration in the gypsum sample over a range of 5–100 mg kg^−1^ using different test kits for higher or lower arsenic contents.

The safety of recycled gypsum powder from the waste plasterboard is essential for various applications, particularly when the gypsum is used in soil. The results of this research are thus expected to be readily applied to the construction of a safe waste plasterboard recycling system that adheres to the concept of a circular economy.

## Data Availability

The data that support the findings of this study are available from the corresponding author, M.T. (Masamoto Tafu), upon reasonable request.
